# Changes in Revolving-Door Mental Health Hospitalizations during the COVID-19 Pandemic: A 5-Year Chart Review Study

**DOI:** 10.3390/jcm12072681

**Published:** 2023-04-04

**Authors:** Giovanni Napoli, Marco Garzitto, Vincenzo Magliulo, Rossana Carnemolla, Calogero Anzallo, Matteo Balestrieri, Marco Colizzi

**Affiliations:** 1 General Hospital Psychiatric Unit, Department of Mental Health, Friuli Centrale Health University Authority, 33100 Udine, Italy; 2 Unit of Psychiatry, Department of Medicine (DAME), University of Udine, 33100 Udine, Italy; 3 Department of Psychosis Studies, Institute of Psychiatry, Psychology and Neuroscience, King’s College London, London SE5 8AF, UK

**Keywords:** mental health care, treatment, care settings

## Abstract

This study assessed changes in revolving-door (RD) mental health hospitalizations during the COVID-19 pandemic. A 5-year retrospective hospital chart review was performed, collecting revolving-door hospitalization, sociodemographic, and clinical data. Out of 1036 patients, 5.69% had RD hospitalizations, which accounted for 10.38% of all recorded hospitalizations. Further, a higher number of RD hospitalizations occurred following the pandemic outbreak, which is unlikely to have been a result of the confounding effect of trimester and month of hospitalization. Finally, several sociodemographic and clinical characteristics recurred more frequently in the context of RD hospitalizations, such as being younger, being compulsorily admitted, being an absconding patient, and being referred by a public service. Certain diagnostic categories occurred more frequently among RD hospitalizations, including psychotic, personality, and substance use disorders, as well as intellectual disability. Patients with specific characteristics are more likely to incur in RD hospitalizations, requiring the implementation of supportive treatment plans, especially following the pandemic outbreak.

## 1. Introduction

Over the last five decades, following the implementation of deinstitutionalizing policies [1], a phenomenon called “revolving-door” (RD) has become evident in mental healthcare [2], with a low number of patients consuming a high proportion of resources due to frequent re-hospitalizations [3]. RD hospital stays are considered a negative outcome, not only in terms of healthcare costs, but especially because of their detrimental effects on patients’ wellbeing and mortality risk [4,5,6]. Despite not being completely clear, RD is considered to be a multifaceted phenomenon, where multiple individuals and environmental and clinical factors come into play [7,8]. While complex clinical presentations may be more difficult to manage, resulting in a per se higher RD risk that is difficult to abate [9,10], the inadequacy of services has been questioned as a potentially addressable RD risk factor [11]. The latter is particularly relevant nowadays since the COVID-19 pandemic has fueled a substantial healthcare system redeployment, often affecting outpatient psychiatric service availability [12] and increasing pressure on acute inpatient services [13,14], with important effects of the pandemic on mental health needs also being documented [15]. Therefore, the aim of this study was to describe the RD phenomenon in a General Hospital Psychiatry Unit (GHPU) in northern Italy, the European epicenter of the pandemic, [13] before and after the onset of the COVID-19 pandemic.

## 2. Materials and Methods

### 2.1. Procedures and Participants

An observational, retrospective chart review study was conducted at the General Hospital Psychiatry Unit (GHPU), Mental Health Department, of the University Hospital of Udine. The GHPU operates across an area of over 500,000 people as the main reference service for mental health emergencies requiring short-term hospitalization, following a mental health assessment at the General Hospital Emergency Department (ED). The main goals of the GHPU are: (i) to stabilize and manage mental health emergencies, (ii) to provide patients with a psychiatric diagnosis, (iii) to begin appropriate treatment, and (iv) to safely discharge patients in continuity with community services, such as Mental Health Services, Addiction Services, Disability Services, and Child/Adolescent Mental Health Services. Information was collected on GHPU admission and discharge dates from the beginning of 2018 to the whole of June 2022. The date of the first Italian COVID-19 case (21 February 2020) was chosen as the onset of the COVID-19 pandemic. All hospitalizations during the observation period were included. A revolving-door (RD) case was defined as presenting with three or more hospitalizations within one year [1,2,16].

### 2.2. Assessment

For each hospitalization, the following information was recorded: (i) patient’s sex (female/male), (ii) ethnicity (Caucasian, Asian, Afro, Hispanic), (iii) age (also described by age-group: <30, 30–40, 41–50, >50 years-old), (iv) history of previous GHPU hospitalization, (v) voluntary/compulsory GHPU hospitalization, (vi) history of previous absconding from the GHPU, (vii) diagnosis according to the tenth International Classification of Diseases (ICD 10; non-affective psychotic disorder, affective disorder, non-psychotic mental disorder, personality disorder, substance use disorder, intellectual disability, physiological condition, other diagnosis), and (viii) referral source (Mental Health Service, Addiction Service, Disability Service, Child/Adolescent Mental Health Service, Private Service).

### 2.3. Statistical Analysis

In preliminary analyses, χ²-, Fisher’s, Welch’s corrected t-, Mann–Whitney’s test, or the analysis of variance were used, as appropriate. In multivariate analyses, categorical measures were included as dummy variables and dimensional measures were standardized in the sample to facilitate comparison of coefficients. As the data collected on the RD phenomenon involved multiple observations per participant and included participants already known to the service at the start date of the observation, they were both left- and right-censored. Thus, to assess RD timing and risk factors, survival analyses with shared frailty were used with a semi-parametric baseline hazard (https://cran.r-project.org/web/packages/frailtyEM/ (accessed on 26 March 2023)). Preliminary observations were evaluated through estimating Fits–Cox’s proportional hazards regression models on both absolute (in days from start of observation) and relative times (in days from first observation in the period of interest) to better disentangle the effect of the COVID-19 onset. Andersen–Gill’s counting process formulation and Kaplan–Meier’s curve were used. Survival probabilities with a 95% confidence interval, coefficient significance (for pre-/post-pandemic comparison), and results of likelihood-ratio test were reported. In analyses with shared frailty, heterogeneity was tested with Commenges–Andersen’s method. Results of the likelihood-ratio test and frailty variance based on γ-distribution with a 95% confidence interval were reported for random effect. Fixed predictors included: (i) COVID-19 pandemic phase, (ii) diagnosis, (iii) age at hospitalization, and being (iv) male, (v) a new GHPU patient, and (vi) being compulsorily admitted (reporting coefficients as odd-ratio with an adjusted 95% confidence interval and z-test with statistical significance). Statistical significance was set at α = 0.050. Analyses were conducted using R-4.2.2 software.

## 3. Results

During the observation period, 1036 patients, 309 of whom were already known to the service, were admitted to the GHPU for a total of 1551 hospitalizations. RD was observed for 59 patients (5.69%) accounting for 161 hospitalizations (10.38%). RD patients were younger than non-RD ones (37.3 ± 14.04 vs. 44.8 ± 15.70 years-old), with more patients in the <30 years-old age-group (40.7% vs. 21.8%) and less in the >50 one (16.9% vs. 37.4%). As expected, they had a higher number of hospitalizations (5.5 ± 2.71 vs. 1.3 ± 0.58) and, consistently had more changes in the diagnoses received upon subsequent hospitalizations (only 27.1% of RD patients presented with a temporal stability of their diagnosis vs 89.7% of non-RD patients). Finally, RD patients had more diagnoses of non-affective psychotic disorder (49.2% vs. 30.4%), personality disorder (33.9% vs. 5.8%), intellectual disability (20.3% vs. 3.8%), substance use disorder (15.3% vs. 7.4%), and other diagnoses (13.6% vs. 3.2%). They also had more compulsory hospitalizations (27.1% vs. 14.1%) and hospitalizations with absconding (28.8% vs. 3.2%; Table 1 and Supplementary Table S1). 

Considering hospitalizations, compared with non-RD ones, those with RD occurred at a younger age (t_205.7_ = 5.40, *p* < 0.001; for age-group: χ^2^_3_ = 58.48, *p* < 0.001) and were more frequently characterized by absconding patients (OR = 3.24, *p* < 0.001). Additionally, during RD hospitalizations, patients were more frequently diagnosed with personality disorder (OR = 6.07, *p* < 0.001) and less frequently diagnosed with affective (OR = 0.47, *p* < 0.001) and non-psychotic mental (OR = 0.51, *p* = 0.005) disorders. Finally, RD hospitalizations were more likely to result from a Mental Health (OR = 1.75, *p* = 0.045) or Addiction Service (OR = 1.83, *p* = 0.048) referral and were less likely to result from a Private Service referral (OR = 0.15, *p* = 0.025). 

During the post-pandemic phase, there were more RD hospitalizations than in the pre-pandemic phase (12.9% vs. 8.0%; OR = 1.70, *p* = 0.002). Additionally, RD frequency was inhomogeneous by year (χ^2^_4_ = 18.53, *p* < 0.001), trimester (χ^2^_3_ = 13.64, *p* = 0.003), and month (χ^2^_11_ = 22.72, *p* = 0.019) of hospitalization, because of more RD hospitalizations in 2021–2022 and in the last months of the year. Instead, no statistically significant interaction was observed between the trimester or month of hospitalization and COVID-19 phase (Supplementary Figures S1 and S2).

**Figure 1:**
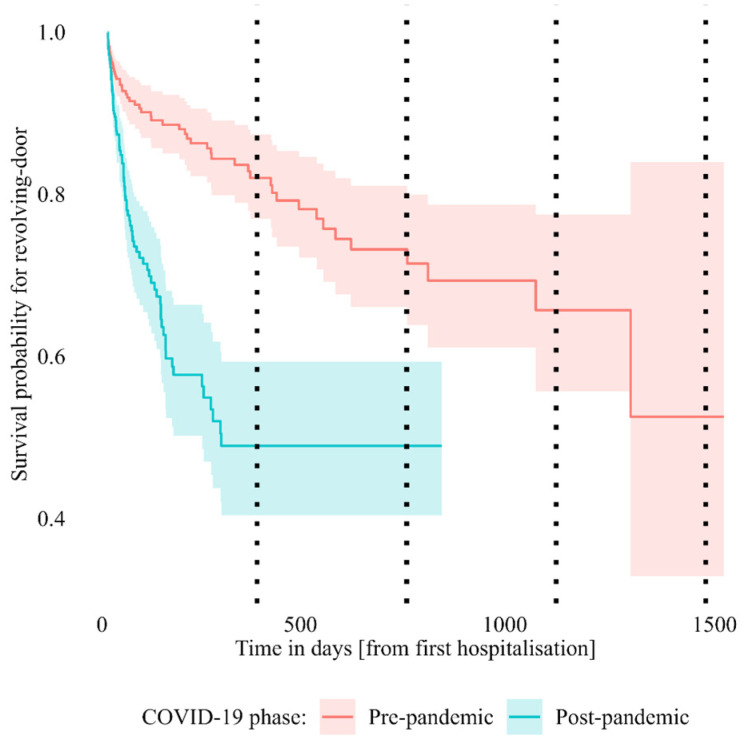
Survival probability for revolving-door in days from the first hospitalization of the patient, before and after the onset of COVID-19 pandemic. Kaplan–Meier’s curve with 95% confidence interval; dotted lines indicate a 1-year interval.

In the whole sample, without corrections, the first RD hospitalization took place at a median of 204 (from 7.8 to 1180.0) days from the first hospitalization. The probability of not showing RD within one year was 0.77 (95% confidence interval: [0.68, 0.88]) and dropped to 0.58 [0.49, 0.69] within two years (Supplementary Figure S3). There was also a statistically significant difference (OR = 2.77, z = +6.04, *p* < 0.001; likelihood-ratio test: 38.03, *p* < 0.01) between pre-pandemic (probability at 1-year: 0.82 [0.77, 0.87]; 2-years: 0.72 [0.64, 0.80]) and post-pandemic (1-year: 0.49 [0.40, 0.59], stable at 2-years) RD hospitalizations, with the latter being more frequent (Figure 1).

When used as a random factor, the trimester (heterogeneity: *p* = 0.283; likelihood-ratio test: *p* = 0.278) and month (*p* = 0.783; *p* > 0.500) of hospitalization were not statistically significant. Furthermore, their regression coefficients were not statistically significant when introduced in a model with the participant as a random factor (all with *p* ≥ 0.200 for trimester and *p* ≥ 0.190 for month). Thus, cyclical effects were not likely to explain the RD phenomenon and were not included in the final model.

In the survival analyses with shared frailty, with the participant as a random factor, participants showed heterogeneity (<0.001) and a statistically significant effect (likelihood-ratio test: *p* < 0.001). Frailty variance was 2.05 [1.01, 3.75]. Being in the post-pandemic phase was associated with a more than twofold risk of RD, while being a new GHPU patient reduced it considerably (Table 2; Supplementary Figures S4 and S5).

## 4. Discussion

To the best of our knowledge, this is the first study investigating the revolving-door (RD) phenomenon focusing on inpatient service use in a general hospital psychiatric unit (GHPU) considering changes that occurred after the COVID-19 pandemic. Other studies have shown a general reduction in the use of inpatient mental health services [17] as well as a reduction in the frequency and duration of psychiatric hospitalizations [15] in the immediate aftermath of the pandemic. Results of this study indicate a relatively low proportion of RD hospitalizations for mental health needs over the study period, although representing a high expenditure to the public health service. Further, a higher number of hospitalizations occurred following the pandemic outbreak, which is unlikely to have been a result of the confounding effect of the trimester and month of hospitalization. Finally, several sociodemographic and clinical characteristics recurred more frequently in the context of RD hospitalizations, such as being younger, being compulsorily admitted, being an absconding patient, and being referred by a public service. A preponderance of certain diagnostic categories was also observed among RD hospitalizations, including psychotic, personality, and substance use disorders as well as intellectual disability. Profiling patients at risk of RD is particularly relevant, as it may help to develop strategies to prevent RD hospitalizations from occurring with the goal of mitigating their health and economic burdens [4,5,6].

The main findings in this study are entirely consistent with previous research reports in suggesting that the RD phenomenon results from the interplay of a wide range of features related to the patient (e.g., early adulthood), the underlying psychiatric disorder (e.g., clinical severity), the treatment plan (e.g., poor healthcare compliance), and the comorbidities (e.g., substance use) [9,10]. With reference to the already suggested role of service availability [11], these results are novel in suggesting more RD hospitalizations since the beginning of the pandemic, possibly reflecting GHPU centrality at a time when other routine services might have been less accessible [13]. Indeed, although community services remained active during the emergency phases of the pandemic, outpatient visits and home care services may have faced significant rescheduling. Such a hypothesis is in line with converging evidence for the long-term effects of the pandemic in increasing access to emergency and acute mental health services [13,18].

Limitations of this study include the collection of data from a single hospital, which thus limits the generalizability of the findings, and the difficulty of comprehensively accounting for the complex clinical profile of patients with acute mental health manifestations. In fact, other information may have been hard to obtain during acute mental healthcare, precluding the investigation of additional risk factors (e.g., patterns of substance use). Finally, apart from recording the disorder for which the patient was hospitalized, more detailed information on the clinical severity was not collected, nor was the reason for RD, which requires investigation in further studies. 

In conclusion, the current results provide a profile of patients likely to incur in RD hospitalizations. Mental healthcare professionals must be aware of the needs of these patients of younger age who are suffering from severe psychiatric disorders and are reluctant to comply with the offered healthcare pathways. This has become particularly relevant over the last few years as the COVID-19 pandemic seems to have caused an increase in RD hospitalization occurrence. More supportive treatment plans for both inpatient- and community-based care should be implemented for such patients and their caregivers.

## Figures and Tables

**Table 1:** Description of participants (N = 1036).

Measure		Without RD	With RD	
Sex	Female	49.5%	45.8%	
	Male	50.5%	54.2%	
Ethnicity	Caucasian	91.8%	86.4%	
	Asian	3.9%	5.1%	
	Afro	2.7%	5.1%	
	Hispanic	1.6%	3.4%	
Age at first hospitalisation		44.8 ± 15.70	37.3 ± 14.04	*
Age-group	<30 years-old	21.8%	40.7%	*
	30–40 years-old	18.8%	18.6%	
	41–50 years-old	22.0%	23.7%	
	>50 years-old	37.4%	16.9%	
Number of hospitalisations		1.3 ± 0.58	5.5 ± 2.71	*
Mean duration of hospitalisation in hours		207.0 ± 403.79	201.0 ± 159.43	
Diagnosis	Psychotic disorder, non-affective	30.4%	49.2%	*
	Affective disorder	32.7%	44.1%	
	Non-psychotic mental disorder	25.6%	35.6%	
	Personality disorder	5.8%	33.9%	*
	Substance use disorder	7.4%	15.3%	*
	Intellectual disability	3.8%	20.3%	*
	Physiological condition	2.1%	5.1%	
	Other diagnosis	3.2%	13.6%	*
Referral source	Mental Health Service	84.2%	91.5%	
	Addiction Service	4.8%	10.2%	
	Disability Service	0.7%	1.7%	
	Child/Adolescent Mental Health Service	0.6%	1.7%	
	Private Service	4.9%	3.4%	
	Unknown Service	8.7%	3.4%	
Any compulsory		14.1%	27.1%	*
Any absconding		3.2%	28.8%	*
Phase of CODID-19	Only pre-pandemic	47.1%	28.8%	*
	Both pre- & post-pandemic	7.6%	45.8%	
	Only post-pandemic	45.3%	25.4%	
Number of RD		-	2.7 ± 2.48	-
Any RD		0.0%	100.0%	-

RD, Hospitalisation classified as revolving-door; *, The difference between patients with and without revolving-door is statistically significant (with *p* < 0.050); percentages are reported for categorical measures; mean and standard deviation are reported for dimensional measures.

**Table 2:** Risk factors for revolving-door hospitalizations.

Predictor	OR [95% CI]	Test
Phase of COVID-19 (post-pandemic = 1)	2.155 [1.403, 3.312]	z = +3.504, *p* < 0.001 *
Relations with the service (new patient = 1)	0.146 [0.076, 0.281]	z = −5.766, *p* < 0.001 *
Kind of hospitalisation (compulsory = 1)	0.939 [0.462, 1.908]	z = −0.175, *p* = 0.861
Psychotic disorder, non-affective (diagnosis = 1)	0.437 [0.153, 1.251]	z = −1.542, *p* = 0.123
Affective disorder (diagnosis = 1)	0.417 [0.143, 1.212]	z = −1.606, *p* = 0.108
Non-psychotic mental disorder (diagnosis = 1)	0.478 [0.164, 1.393]	z = −1.352, *p* = 0.176
Personality disorder (diagnosis = 1)	1.439 [0.505, 4.100]	z = +0.680, *p* = 0.496
Substance abuse disorder (diagnosis = 1)	0.570 [0.165, 1.971]	z = −0.888, *p* = 0.374
Intellectual disability (diagnosis = 1)	0.289 [0.082, 1.022]	z = −1.926, *p* = 0.054
Physiological condition (diagnosis = 1)	0.555 [0.055, 5.567]	z = −0.500, *p* = 0.617
Sex (male = 1)	1.358 [0.793, 2.326]	z = +1.115, *p* = 0.265
Age at the hospitalisation (standardized)	0.802 [0.596, 1.079]	z = −1.459, *p* = 0.144

OR, Odd-ratio; CI, Adjusted confidence interval; *, Statistically significant with *p* < 0.050; regression coefficients for fixed predictors in survival analyses with shared frailty are reported (with participant as random factor).

## Data Availability

The data presented in this study are available upon request from the corresponding author.

## Supplementary Materials

The following supporting information can be downloaded at: https://www.mdpi.com/article/10.3390/jcm12072681/s1, Table S1: Description of participants (N = 1036); Figure S1: Distribution of the proportion of hospitalizations classified as revolving-door by year, trimester, and month; Figure S2: Distribution of the proportion of hospitalizations classified as revolving-door by year, trimester, and month, considering the phase of COVID-19 pandemic (pre- and post-onset in Italy, 21 February 2020); Figure S3: General description of observations; Figure S4: Cumulative hazard for revolving-door in patients at first contact with service, before and after COVID-19 onset; Figure S5: Cumulative hazard for revolving-door in a previously hospitalized patient, before and after COVID-19 onset.
