# Supplementary material for: Changes in Revolving-Door Mental Health Hospitalizations during the COVID-19 Pandemic: A 5-Year Chart Review Study
Source: J Clin Med. 2023 Apr 4;12(7):2681. doi: 10.3390/jcm12072681 (PMC10095521; doi:10.3390/jcm12072681)
Supplement: Supplementary file 1 [file jcm-12-02681-s001.zip › jcm-2274769-supplementary.pdf]

Communication

Changes in Revolving-Door Mental Health Hospitalizations during the COVID-19 Pandemic: A 5-Year Chart Review Study

Supplementary Table S1. Description of participants (N=1,036).

Measure		Participants without RD	Participants with RD	Comparison between Participants without/with RD
Sex	*Female*	484 (49.5%)	27 (45.8%)	OR=1.16 (0.664, 2.052), p=0.594
	Male	493 (50.5%)	32 (54.2%)	
Ethnicity	*Caucasian*	897 (91.8%)	51 (86.4%)	$\chi^2_{df=4}$=2.53, p=0.470
	Asian	38 (3.9%)	3 (5.1%)	
	Afro	26 (2.7%)	3 (5.1%)	
	Hispanic	16 (1.6%)	2 (3.4%)	
Age at first hospitalisation		44.8 ±15.70 [17, 85]	37.3 ±14.04 [17, 75]	$t_{df=67.1}$=+3.96, p<0.001*
Age-group	*<30 years-old*	213 (21.8%)	24 (40.7%)	$\chi^2_{df=3}$=15.15, p=0.002*
	30-40 years-old	184 (18.8%)	11 (18.6%)	
	41-50 years-old	215 (22.0%)	14 (23.7%)	
	>50 years-old	365 (37.4%)	10 (16.9%)	

Measure		Participants without RD	Participants with RD	Comparison between Participants without/with RD
Number of hospitalisations		1.3 ±0.58 [1, 6]	5.5 ±2.71 [2, 19]	U=507.0, $p<0.001$*
Mean duration of hospitalisation in hours		207.0 ±403.79 [1.0, 7176.0]	201.0 ±159.43 [12.9, 794.5]	$t_{df=110.7}=+0.25$, p=0.806
Diagnosis	*Psychotic disorder, non-affective*	297 (30.4%)	29 (49.2%)	OR=2.21, 95% CI: (1.26, 3.89), p=0.004*
	Affective disorder	319 (32.7%)	26 (44.1%)	OR=1.62, 95% CI: (0.92, 2.85), p=0.087
	Non-psychotic mental disorder	250 (25.6%)	21 (35.6%)	OR=1.61, 95% CI: (0.88, 2.87), p=0.095
	Personality disorder	57 (5.8%)	20 (33.9%)	OR=8.24, 95% CI: (4.27, 15.60), p<0.001*
	Substance use disorder	72 (7.4%)	9 (15.3%)	OR=2.26, 95% CI: (0.94, 4.89), p=0.041*
	Intellectual disability	37 (3.8%)	12 (20.3%)	OR=6.46, 95% CI: (2.88, 13.71), p<0.001*
	Physiological condition	21 (2.1%)	3 (5.1%)	OR=2.44, 95% CI: (0.45, 8.53), p=0.152
	Other diagnosis	31 (3.2%)	8 (13.6%)	OR=4.77, 95% CI: (1.80, 11.32), p=0.001*
Referral source	*Mental Health Service*	823 (84.2%)	54 (91.5%)	OR=2.02, 95% CI: (0.80, 6.58), p=0.190
	Addiction Service	47 (4.8%)	6 (10.2%)	OR=2.24, 95% CI: (0.75, 5.58), p=0.116
	Disability Service	7 (0.7%)	1 (1.7%)	OR=2.39, 95% CI: (0.05, 19.08), p=0.375
	Child/Adolescent Mental Health Service	6 (0.6%)	1 (1.7%)	OR=2.79, 95% CI: (0.06, 23.55), p=0.337
	Private Service	48 (4.9%)	2 (3.4%)	OR=0.68, 95% CI: (0.08, 2.71), p>0.999
	Unknown Service	85 (8.7%)	2 (3.4%)	OR=0.37, 95% CI: (0.04, 1.44), p=0.223
Any compulsory		138 (14.1%)	16 (27.1%)	OR=2.26, 95% CI: (1.16, 4.23), p=0.013*
Any absconding		31 (3.2%)	17 (28.8%)	OR=12.28, 95% CI: (5.89, 25.06), p<0.001*

Measure		Participants without RD	Participants with RD	Comparison between Participants without/with RD
Phase of CODID-19	*Only pre-pandemic*	460 (47.1%)	17 (28.8%)	
	Both pre- & post-pandemic	74 (7.6%)	27 (45.8%)	$\chi^2_{df=2}$=92.26, p<0.001*
	Only post-pandemic	443 (45.3%)	15 (25.4%)	
Number of RD		-	2.7 ±2.48 [1, 17]	-
Any RD		0 (0.0%)	59 (100.0%)	-

CI, Confidence interval; df, Degrees of freedom; RD, Hospitalisation classified as revolving-door; OR, Odd-ratio; *, The difference between patients with and without revolving-door is statistically significant (with p<0.050); frequencies and percentage (between brackets) are reported for categorical measures; mean, standard deviation, and range (between square brackets) are reported for dimensional measures

Supplementary Figure S1. Distribution of the proportion of hospitalizations classified as revolving-door by year, trimester, and month.

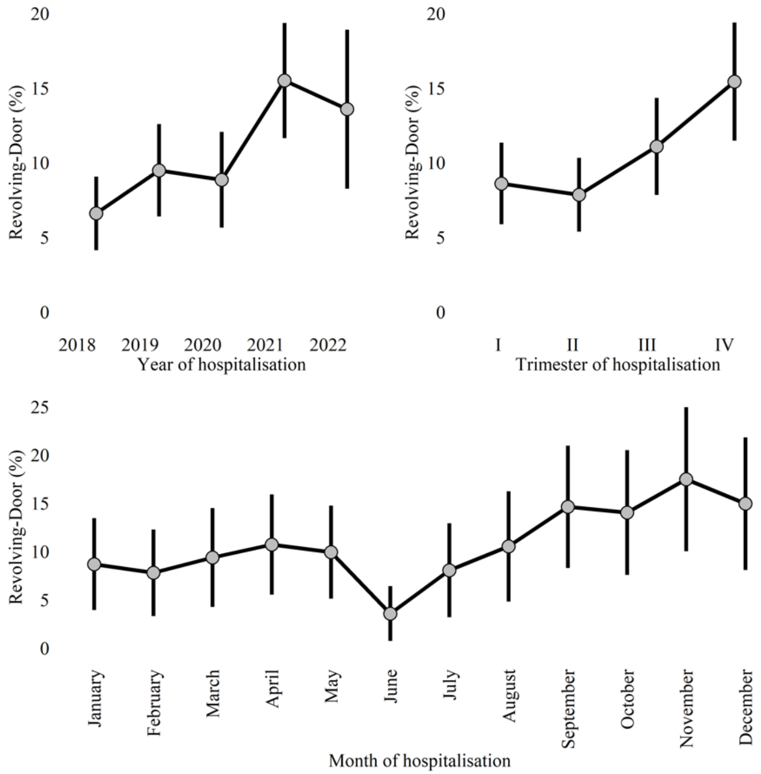

	**Test of main effect**
Year of hospitalization	$F_{4,1546}$=+4.67, p<0.001***
Trimester of hospitalization	$F_{3,1547}$=+4.58, p=0.003**
Month of hospitalization	$F_{11,1539}$=+2.08, p=0.019*

Supplementary Figure S2. Distribution of the proportion of hospitalizations classified as revolving-door by year, trimester, and month, considering the phase of COVID-19 pandemic (pre- and post-onset in Italy, February 21, 2020).

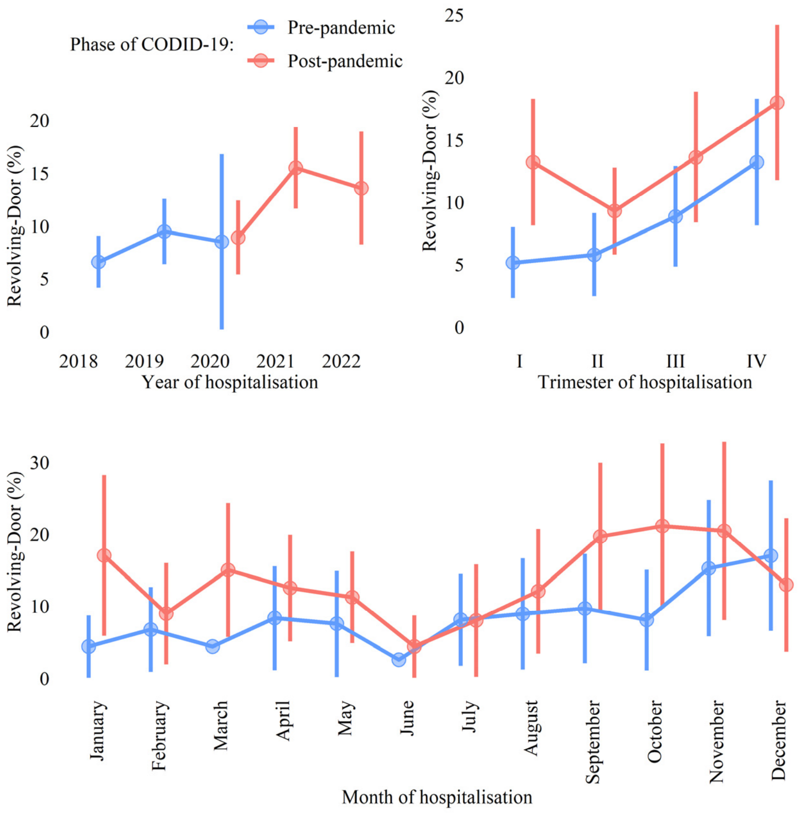

	**Test of interaction**
Trimester of hospitalization by phase of COVID-19	$F_{3,1543}$=+0.42, p=0.736
Month of hospitalization by phase of COVID-19	$F_{11,1527}$=+0.92, p=0.524

Supplementary Figure S3. General description of observations.

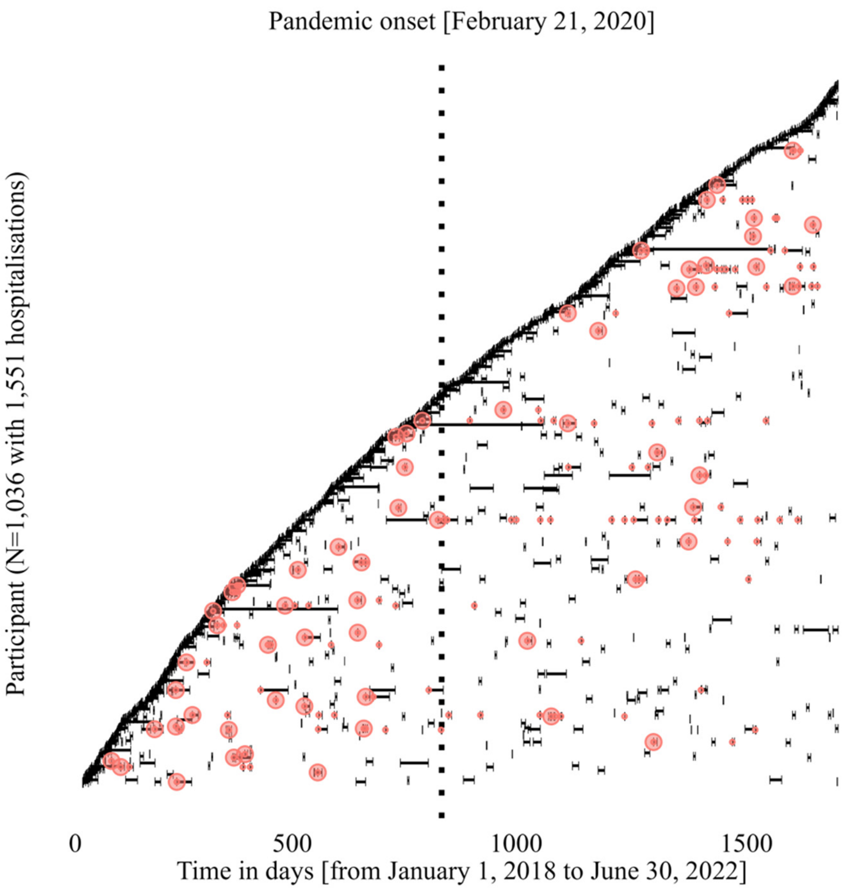

Hospitalizations organized by participant (with duration in days). Hospitalizations classified as revolving-door are marked (circles), with larger circles identifying the first hospitalization with revolving-door for the participant. The onset of COVID-19 pandemic in Italy is indicated by a dotted vertical line

Supplementary Figure S4. Cumulative hazard for revolving-door in patients at first contact with service, before and after COVID-19 onset

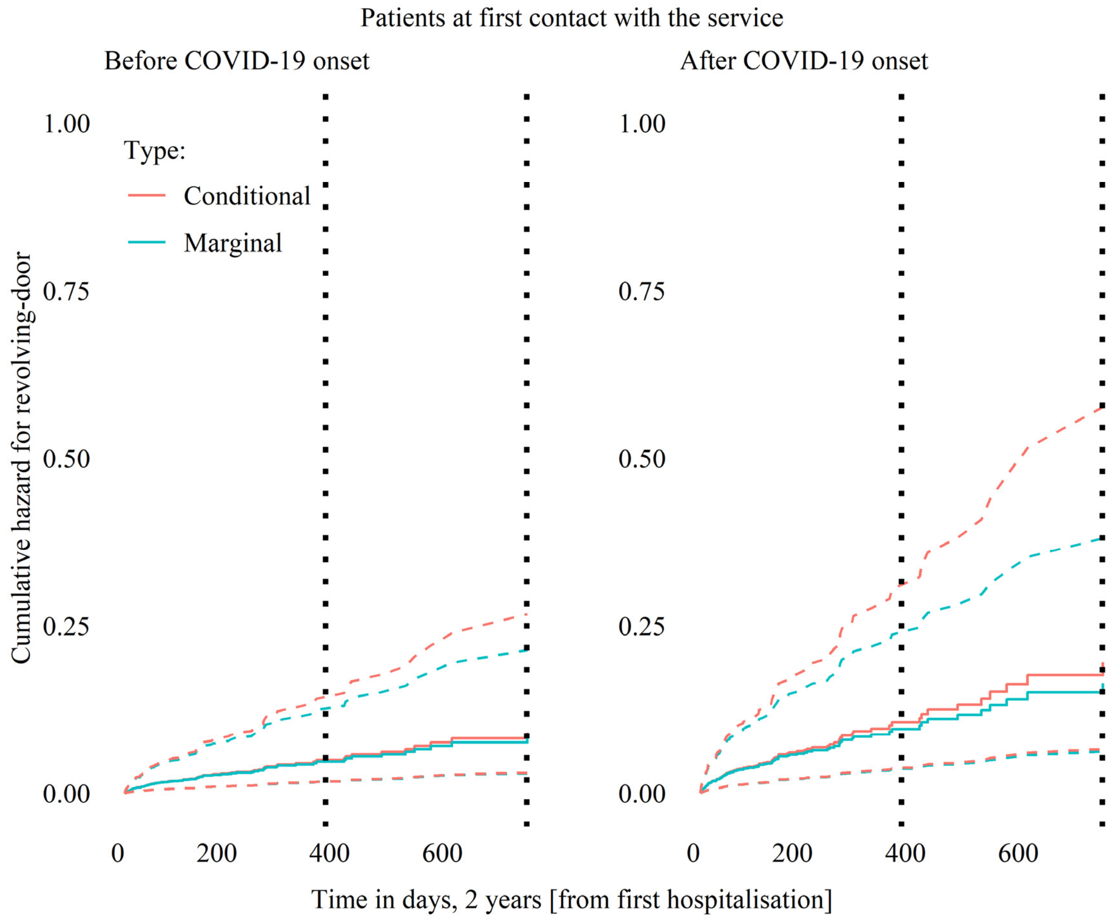

Survival analysis with shared frailty; both marginal and conditional cumulative hazards are reported; models are fitted for a prevision of 2-years from the first hospitalization, with dotted lines indicating years

Supplementary Figure S5. Cumulative hazard for revolving-door in a previously hospitalized patient, before and after COVID-19 onset

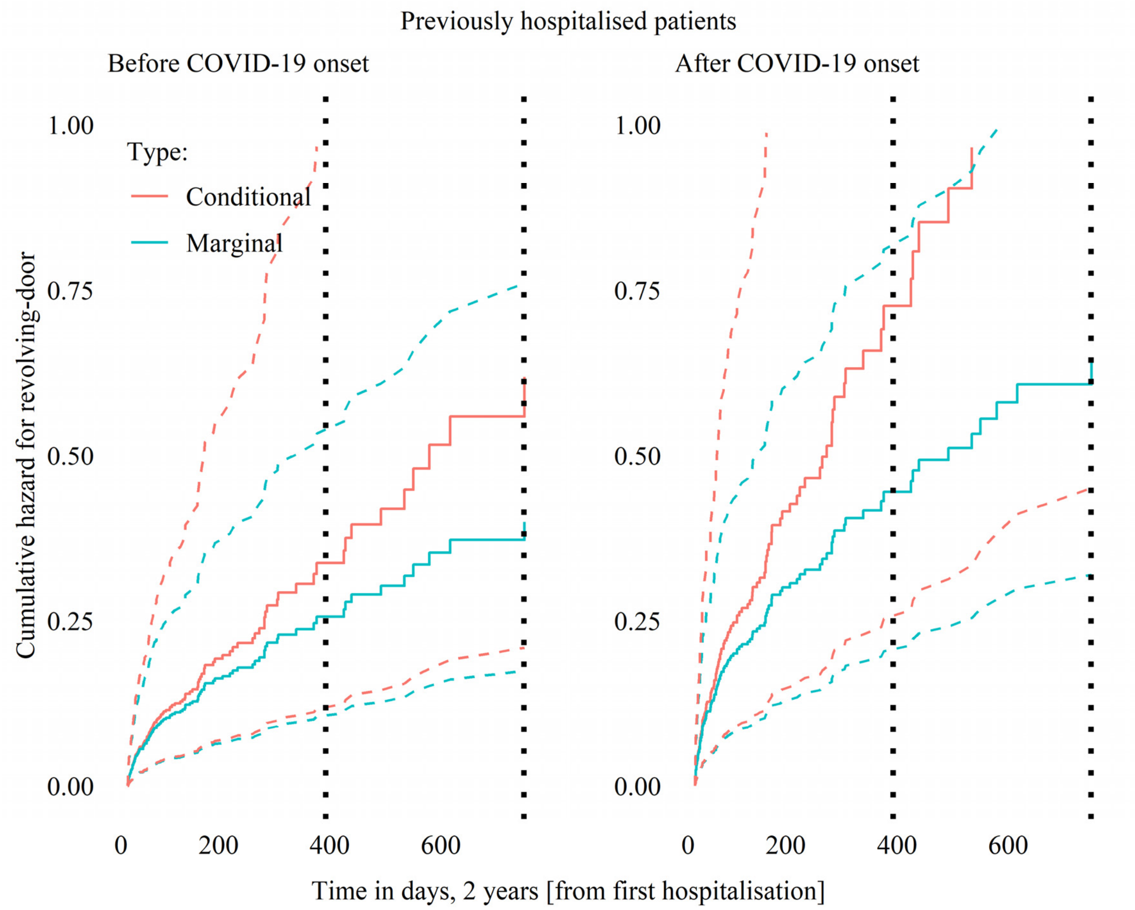

Survival analysis with shared frailty; both marginal and conditional cumulative hazards are reported; models are fitted for a prevision of 2-years from the first hospitalization, with dotted lines indicating years
